# The underestimated risk of additional ipsilateral BI-RADS 3 lesions identified by preoperative ultrasound in breast cancer patients

**DOI:** 10.1186/s13244-026-02366-6

**Published:** 2026-10-16

**Authors:** Yue Hu, Hongli Wang, Mengzi Wu, Wei Zheng, Jingsi Mei, Xiaofang Jiang, Queran Lin, Ran Gu, Fengtao Liu, Jinna Lin, Chang Gong, Jun Shen, Qiang Liu

**Affiliations:** 1 Breast Tumor Center, Sun Yat-Sen Memorial Hospital, Sun Yat-Sen University, Guangzhou, P.R. China; 2 Guangdong Provincial Key Laboratory of Malignant Tumor Epigenetics and Gene Regulation, Medical Research Center, Sun Yat-Sen Memorial Hospital, Sun Yat-Sen University, Guangzhou, P.R. China; 3 Department of Radiology, Sun Yat-Sen Memorial Hospital, Sun Yat-Sen University, Guangzhou, P.R. China

**Keywords:** Ultrasonography, BI-RADS category 3, Breast neoplasms

## Abstract

**Objective:**

To determine the prevalence of malignancy of additional ultrasound BI-RADS category 3 lesions in women with newly diagnosed breast cancer.

**Materials and methods:**

Between July 2014 and June 2022, women with newly diagnosed breast cancer and additional ultrasound BI-RADS 3 lesions were retrospectively included. Malignancy was confirmed by pathology, and malignancy rates were assessed at both the patient and lesion levels. Independent risk factors for malignant BI-RADS 3 lesions were identified using multivariable logistic regression.

**Results:**

A total of 1458 women (median age, 45 years; IQR: 40–51) with 1995 ultrasound BI-RADS 3 lesions were included. At the patient level, malignancy was identified in 3.5% of patients (51/1458), with higher rates for ipsilateral than contralateral BI-RADS 3 lesions (9.8% [39/397] vs 1.1% [12/1061]; *p* < 0.001). At the lesion level, the overall malignancy rate was 2.7% (54/1995), similarly elevated for ipsilateral compared with contralateral lesions (7.5% [41/544] vs 0.9% [13/1451]; *p* < 0.001). Independent predictors for malignancy of ipsilateral BI-RADS 3 lesions included same-quadrant location as the primary lesion (OR, 4.3; 95% CI: 1.9, 9.7; *p* < 0.001), ultrasonographic architectural distortion of the primary lesion (OR, 8.5; 95% CI: 1.6, 46.1; *p* = 0.01), and mammographic suspicious calcification of the primary lesion (OR, 2.9; 95% CI: 1.4, 6.0; *p* = 0.004).

**Conclusion:**

Additional ipsilateral ultrasound BI-RADS 3 lesions in women with newly diagnosed breast cancer demonstrated malignancy rates exceeding the 2% BI-RADS threshold, particularly when located in the same quadrant as the primary cancer.

**Key Points:**

***Question*** The malignancy risk of additional BI-RADS 3 lesions identified by preoperative ultrasound in breast cancer patients remains unclear.

***Findings*** Ipsilateral lesions, especially in the same quadrant, show malignancy rates exceeding 2%, while malignancy risk remains low for contralateral BI-RADS 3 lesions.

***Critical relevance statement*** Ipsilateral BI-RADS 3 lesions in the same quadrant as the primary cancer, or those with primary tumor architectural distortion or suspicious calcifications, warrant biopsy. This ensures precise surgical planning and avoids missing additional malignancy.

**Graphical Abstract:**

**Figure:**
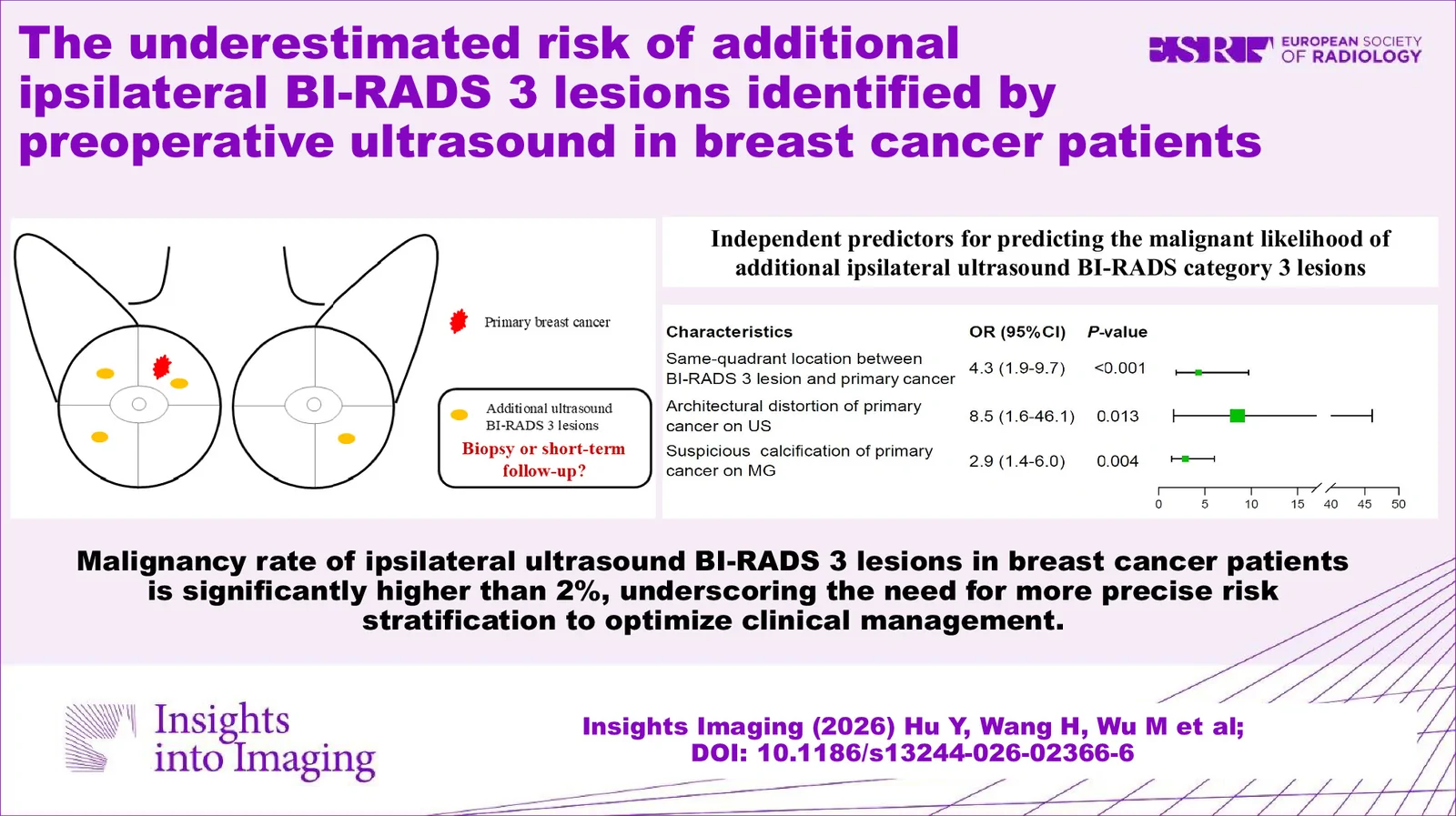

## Introduction

Synchronous ipsilateral or contralateral breast cancers are frequently observed in patients with newly diagnosed breast cancer [1, 2]. Reported prevalence of multiple malignant foci in the ipsilateral breast ranges widely from 9% to 75%, and synchronous bilateral breast cancer has been reported in 1.4%–2.9% of cases [1–4]. Thus, accurate identification of malignancy in additional breast lesions is crucial for making the optimal treatment decision in patients with breast cancer.

Ultrasound has been increasingly used to screen for potential additional lesions in women with newly diagnosed breast cancer [5–7]. If an additional lesion is detected and classified as Breast Imaging Reporting and Data System (BI-RADS) category 3 according to the American College of Radiology (ACR), this lesion is deemed probably benign with a malignancy likelihood of less than 2% and is recommended to undergo short-term follow-up rather than biopsy [8]. However, in the preoperative setting, this approach may lead to diagnostic uncertainty, increased patient anxiety, and possible repeat biopsy or re-excision during follow-up. Therefore, clarifying the actual malignancy risk of these additional BI-RADS 3 lesions is essential for preoperative decision-making. Previous studies have indicated that the malignancy rates of additional ultrasound BI-RADS category 3 lesions in women with newly diagnosed breast cancer may significantly exceed the standard 2% threshold [9, 10]. However, these prior studies were limited by relatively small patient cohorts or short-term imaging follow-up. Therefore, the actual risk of malignancy of additional ultrasound BI-RADS 3 in women with newly diagnosed breast cancer remains to be adequately determined.

In this study, women with newly diagnosed breast cancer who had additional ipsilateral or contralateral ultrasound BI-RADS 3 lesions based on the 2013 BI-RADS standard were retrospectively included. The primary purpose of this study was to determine the prevalence of malignancy of these additional ultrasound BI-RADS 3 lesions.

## Materials and methods

This retrospective observational study was approved by the institutional review board of Sun Yat-Sen Memorial Hospital of Sun Yat-Sen University (registration no. SYSKY-2024-510-01), and the requirement for informed consent was waived due to the nature of the study.

### Patients

**Fig. 1:**
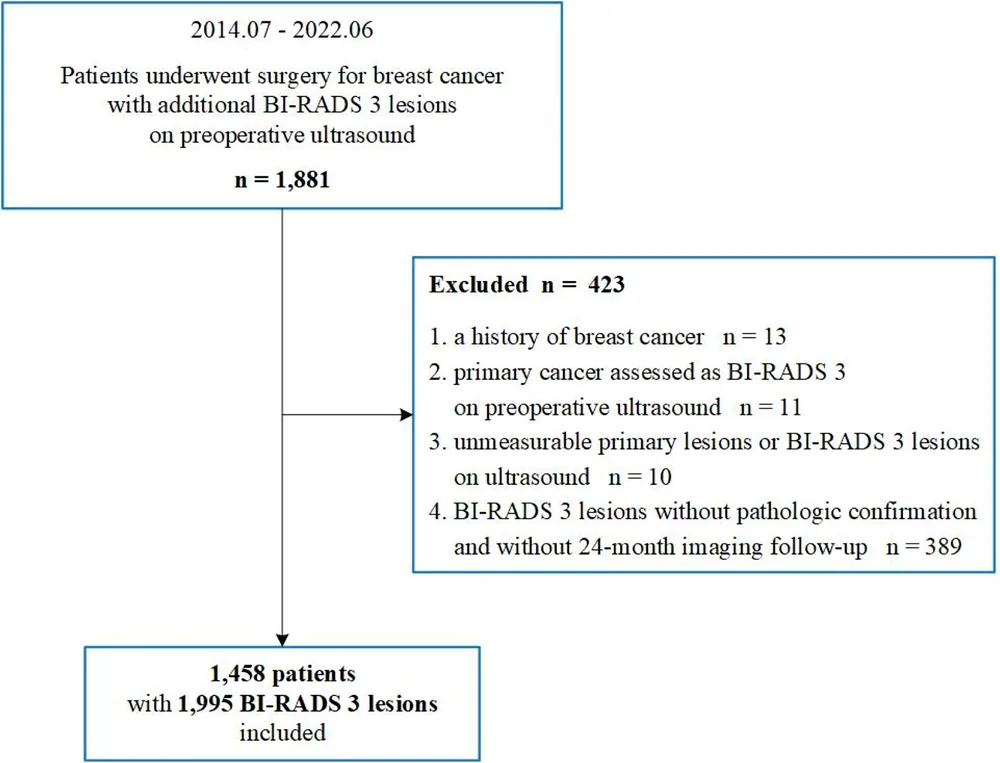
Flowchart of patient selection. BI-RADS, breast imaging reporting and data system

Women with newly diagnosed breast cancer who underwent surgical treatment at our institution between July 2014 and June 2022 were retrospectively identified from the electronic medical record system. The inclusion criteria were as follows: (1) patients with newly diagnosed breast cancer (primary lesion); (2) other ipsilateral or contralateral lesions on preoperative breast ultrasound assessed as BI-RADS 3 (additional ultrasound BI-RADS 3 lesions). The exclusion criteria were as follows: (1) a history of breast cancer; (2) primary lesion assessed as BI-RADS category 3 on preoperative breast ultrasound; (3) unmeasurable primary or BI-RADS 3 lesions on ultrasound, e.g. duct changes or calcifications without a mass; (4) ultrasound BI-RADS 3 lesions lacking pathologic confirmation and those without at least 24 months of imaging follow-up to confirm the absence of progression, most commonly attributable to: (i) excision together with the primary tumor for ultrasound BI-RADS 3 lesions located within 2 cm of it, (ii) therapeutic or prophylactic mastectomy, or (iii) patient loss to follow-up. Therefore, the final analysis was restricted to BI-RADS 3 lesions located ≥ 2 cm from the primary lesion, which had either pathologic or imaging follow-up data available. The flowchart of patient selection is shown in Fig. 1.

### Ultrasound examination

**Fig. 2:**
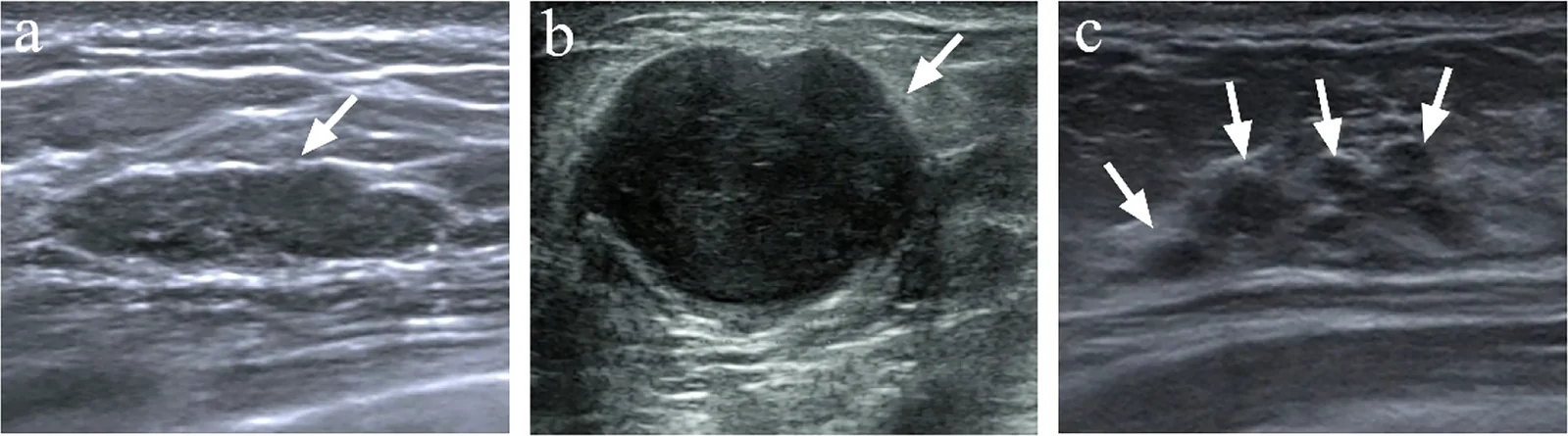
Representative ultrasound images of lesions with typical BI-RADS category 3 features. **a** A solid oval hypoechoic mass with a circumscribed margin and parallel orientation. **b** An isolated complicated cyst. **c** Clustered microcysts. BI-RADS = Breast Imaging Reporting and Data System

Breast ultrasound examinations were performed by one of six breast radiologists (Y.H., H.W., J.M., X.J., R.G., and F.L.; each with more than 5 years’ experience of breast ultrasound). All patients routinely underwent bilateral whole-breast ultrasound examination as part of their preoperative imaging assessment. Each examination was performed with a high-resolution unit (S2000, S1000 or ACUSON Oxana2, Siemens Medical Solutions; RS80A, Samsung Medison; LOGIQ 10S, GE HealthCare) with a high-frequency linear transducer. Representative images were documented, and a final category was assigned to each lesion according to the 2013 ACR BI-RADS standard [8]. Specifically, a lesion was classified as BI-RADS category 3 if it presented ultrasonographic features such as a solid mass with circumscribed margin, oval shape, and parallel orientation; an isolated complicated cyst; or clustered microcysts. Fig. 2 presents representative examples of lesions demonstrating these typical BI-RADS 3 characteristics. Two senior radiologists (Y.H. and H.W., with 15 and 9 years of experience in breast ultrasound, respectively) retrospectively assessed the ultrasonographic features independently and confirmed the BI-RADS category 3 assessment according to the 2013 ACR BI-RADS standard [8]. Any disagreements were resolved in consensus.

For cases with multiple primary cancers in the unilateral breast, the largest one was selected. The following characteristics of the primary cancer were recorded: BI-RADS assessment, maximum diameter, and ultrasonographic features, including shape (oval, round, or irregular), orientation (parallel or not parallel), margin (circumscribed or not circumscribed), echo pattern (hyperechoic, mixed solid and cystic, hypoechoic, isoechoic, or heterogeneous) and posterior features (no posterior features, enhancement, shadowing, or combined pattern); the presence of calcification (yes or no); associated features such as architectural distortion (yes or no), duct changes (yes or no), skin changes (yes or no), edema (yes or no) and vascularity (absent, internal vascularity, or vessels in rim); as well as the suspicious status of axillary lymph node (yes or no).

All eligible additional ultrasound BI-RADS 3 lesions were recorded for subsequent analyses, including their maximum diameter and anatomical relationship to the primary tumor, defined as located in the same quadrant, a different quadrant of the ipsilateral breast, or the contralateral breast. To ensure consistency in the patient-level analysis, a single representative BI-RADS 3 lesion was selected for each patient. This representative lesion was chosen based on the following criteria: (1) if any malignancy was present, the pathologically confirmed malignant lesion was selected; (2) in cases with multiple lesions of the same pathologic nature, the one with the largest maximum diameter was chosen. The reasons for underestimation of each malignant BI-RADS 3 lesion were also documented by referencing the criteria described previously [11].

### Mammography examination

Bilateral digital mammography with two standard imaging planes (mediolateral oblique and craniocaudal) was performed using a digital mammographic unit (Planmed, Helsinki, Finland; Hologic Inc.). Images were reviewed retrospectively by two senior breast radiologists (Y.H. and H.W.), and any disagreements were resolved in consensus. The presence or absence of suspicious calcification of the primary breast cancer was recorded. Mammographic images were also reviewed to evaluate whether any findings corresponded to the ultrasound BI-RADS 3 lesions.

### Breast MRI

Preoperative breast MRI was not routinely performed during the study period. For patients who underwent MRI, images were retrospectively reviewed to assess whether any findings corresponded to the ultrasound BI-RADS 3 lesions.

### Pathology

Malignancy was confirmed by pathology. BI-RADS 3 lesions were biopsied using a vacuum-assisted biopsy (VAB) device under real-time ultrasound guidance with an 8-gauge or 11-gauge needle, or alternatively, excised surgically for pathologic diagnosis. In our clinical practice, VAB was routinely used for most BI-RADS 3 lesions because of its higher diagnostic accuracy, ability to obtain larger tissue samples, and potential for complete excision of benign lesions [12, 13]. VAB was generally preferred for lesions < 2 cm that were clearly visible on ultrasound and located away from the nipple or chest wall. In contrast, open surgery was typically performed for larger lesions (> 2 cm) or lesions not amenable to percutaneous biopsy; notably, the final modality was individualized based on surgeon discretion and patient preference. The criteria for pathologic examinations were based on the World Health Organization classification of breast tumors [14]. The molecular subtypes were identified according to the 2013 St Gallen consensus [15].

### Statistical analysis

Statistical analyses were performed at two distinct levels. At the lesion level, all additional ultrasound BI-RADS 3 lesions were included to evaluate the overall malignancy risk of BI-RADS 3 categorization. At the patient level, only the representative lesion per patient was included to calculate per-person malignancy rates and to identify independent risk factors. Quantitative variables were expressed as the median and interquartile range (IQR), while qualitative variables were summarized as frequencies and percentages. The Pearson χ^2^ test or Fisher exact test was utilized to compare qualitative variables. Logistic regression analysis was performed at the patient level to identify independent predictors of malignant BI-RADS 3 lesions. Variables with a significance level of *p* < 0.05 in univariable analysis were included in the multivariable logistic regression analysis using enter selection. Variance inflation factors and tolerances were calculated to assess the collinearity assumption, with a variance inflation factor less than 10 and a tolerance greater than 0.1 indicating no significant collinearity. A prediction model was developed based on significant predictors identified in the multivariable logistic regression analysis. Its predictive performance was assessed using area under the receiver-operating characteristic curve. SPSS software (version 22.0; IBM SPSS), Medcalc software (version 22.001; Medcalc Software) and R software (version 4.1.3; R Development Core Team) were used for the statistical analysis. All *p*-values were two-tailed, and *p* < 0.05 was considered to indicate statistical significance.

## Results

### Clinical characteristics of patients

A total of 1458 women with newly diagnosed breast cancer and additional ultrasound BI-RADS 3 lesions were included in the study. The median age was 45 years (IQR, 40–51 years). The median size of the primary tumors was 2.4 cm (range, 0.4–10.4 cm), whereas that of all identified BI-RADS 3 lesions was 0.8 cm (range, 0.3–7.5 cm).

At the patient level, 902 of 1458 patients (61.9%) underwent biopsy of their representative ultrasound BI-RADS 3 lesion, whereas the remaining 556 patients (38.1%) were managed with imaging follow-up for at least 24 months. In total, 397 patients had ipsilateral BI-RADS 3 lesions, including 177 located in the same quadrant and 220 in a different quadrant relative to the primary tumor, while 1061 patients had contralateral lesions.

At the lesion level, a total of 1,995 ultrasound BI-RADS 3 lesions were identified, with 1184 (59.3%) undergoing biopsy and 811 (40.7%) receiving imaging follow-up. Among the biopsied lesions, 943 (79.6%) were sampled using VAB, while 241 (20.4%) were removed via open surgical excision. Of all BI-RADS 3 lesions, 544 were ipsilateral (235 in the same quadrant and 309 in a different quadrant), while 1451 were located in the contralateral breast relative to the primary tumor. The distribution of additional BI-RADS 3 lesions at both the patient and lesion levels is summarized in Table 1.

**Table 1:** Distribution and malignancy rates of additional ultrasound BI-RADS category 3 lesions at the patient and lesion levels

Level	Location	Total (*n*)	Malignant (*n*)	Malignancy rate (%)
Patient-level	Overall	1458	51	3.5
	Ipsilateral	397	39	9.8
	Same quadrant	177	30	16.9
	Different quadrant	220	9	4.1
	Contralateral	1061	12	1.1
Lesion-level	Overall	1995	54	2.7
	Ipsilateral	544	41	7.5
	Same quadrant	235	32	13.6
	Different quadrant	309	9	2.9
	Contralateral	1451	13	0.9

### Likelihood of malignancy of additional ultrasound BI-RADS 3 lesions

At the patient level, 51 patients had additional ultrasound BI-RADS 3 lesions confirmed as malignant, yielding an overall malignancy rate of 3.5% (51/1458). Detailed malignancy rates are provided in Table 1. Patients with ipsilateral BI-RADS 3 lesions had a significantly higher malignancy rate of 9.8% (39/397; 95% CI: 7.1%–13.2%), compared with 1.1% (12/1061; 95% CI: 0.6%–2.0%) in those with contralateral BI-RADS 3 lesions (*p* < 0.001). Within the ipsilateral group, lesions located in the same quadrant as the primary tumor showed a substantially higher malignancy rate than those in a different quadrant (16.9% [30/177; 95% CI, 11.7%–23.3%] vs 4.1% [9/220; 95% CI, 1.9%–7.6%], *p* < 0.001).

At the lesion level, a total of 1,995 ultrasound BI-RADS 3 lesions were identified, among which 54 were malignant, resulting in an overall malignancy rate of 2.7% (54/1,995; 95% CI: 2.0%–3.5%). Of these 1995 lesions, 26 (1.3%) were characterized as clustered microcysts. Notably, all clustered microcysts in this study were confirmed to be benign through histopathology or stable imaging follow-up for at least 24 months. Among the 544 ipsilateral BI-RADS 3 lesions, 41 were malignant (7.5%; 95% CI: 5.5%–10.1%), whereas contralateral lesions showed a significantly lower malignancy rate (13/1,451; 0.9%; 95% CI: 0.5%–1.5%, *p* < 0.001). Further stratification revealed that ipsilateral BI-RADS 3 lesions located in the same quadrant as the primary tumor had a malignancy rate of 13.6% (32/235; 95% CI: 9.5%–18.6%), while those in a different quadrant showed a rate of 2.9% (9/309; 95% CI: 1.3%–5.5%, *p* < 0.001).

Of the 1458 patients in the study population, 1442 (98.9%) underwent preoperative mammography, and 557 (38.2%) underwent breast MRI. Among the 51 patients with confirmed malignant additional ultrasound BI-RADS 3 lesions, 50 (98.0%) had preoperative mammography, of which 46 (92.0%) were radiologically occult; the remaining 4 cases showed non-specific findings including corresponding masses (*n* = 3) or focal asymmetry (*n* = 1). Additionally, 16 (31.4%) of these 51 patients received preoperative MRI, with 6 (37.5%) being radiologically occult and the others manifesting as non-specific foci (*n* = 5), masses (*n* = 3), or non-mass enhancement (*n* = 2).

Given that the malignancy rate of additional BI-RADS 3 lesions located in the ipsilateral breast exceeded the 2% threshold, subsequent analyses were restricted to the 397 cases with ipsilateral BI-RADS 3 lesions and conducted at the patient level.

### Risk factors for malignant ipsilateral ultrasound BI-RADS 3 lesions

**Fig. 3:**
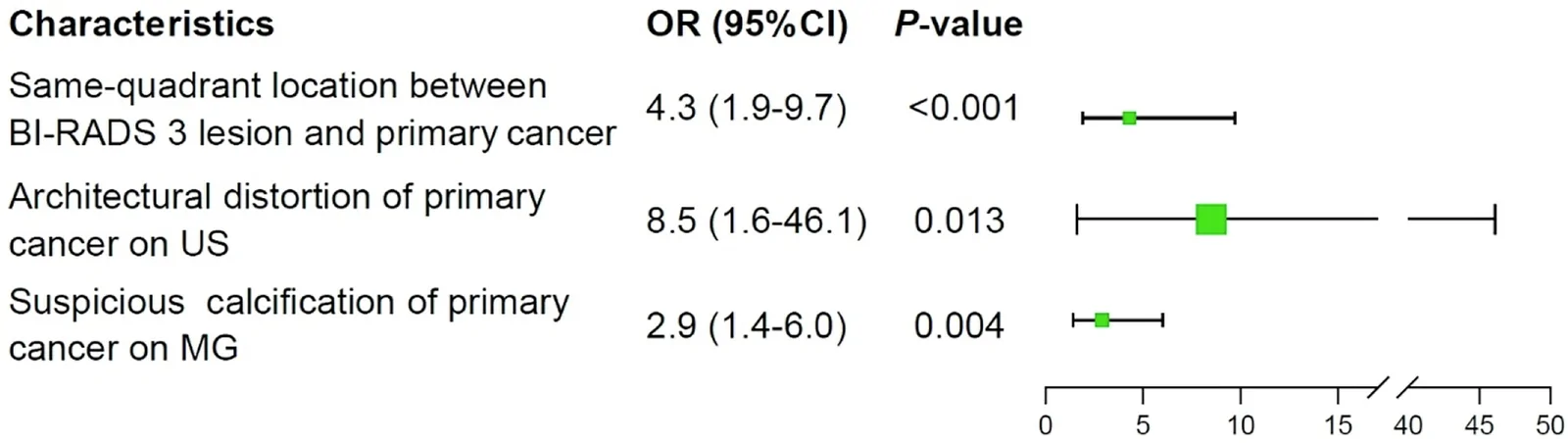
Forest plot shows independent predictors for predicting the malignant likelihood of additional ipsilateral ultrasound BI-RADS category 3 lesions. Green boxes indicate OR, and the line segments indicate 95% CI. BI-RADS, breast imaging reporting and data system; US, ultrasound; MG, mammography

Detailed characteristics of cases with ipsilateral ultrasound BI-RADS 3 lesions are presented in Tables 2 and 3. Univariable logistic regression analysis showed that the anatomical relationship between the BI-RADS 3 lesions and primary cancer (*p* < 0.001), architectural distortion of primary cancer on ultrasound (*p* = 0.03), and suspicious calcification of primary cancer on mammography (*p* = 0.002) were associated with the malignancy of additional BI-RADS 3 lesions. In contrast, no significant associations were observed between the malignancy of these lesions and the primary tumor’s biological or clinical characteristics, including intrinsic subtype (*p* = 0.15), pathologic type (*p* = 0.32), and patient age (*p* = 0.07). Further multivariable analysis showed that the same-quadrant location as the primary cancer (odds ratio [OR], 4.3; 95% CI: 1.9, 9.7; *p* < 0.001), architectural distortion of primary cancer on ultrasound (OR, 8.5; 95% CI: 1.6, 46.1; *p* = 0.01), and suspicious calcification of primary cancer on mammography (OR, 2.9; 95% CI: 1.4, 6.0; *p* = 0.004) were independent predictors (Fig. 3). All the risk factors selected by the multivariable logistic regression analysis had a variance inflation factor less than 10 and tolerance greater than 0.1, suggesting no apparent collinearity among them. A prediction model combining these three risk factors yielded an area under the receiver-operating characteristic curve of 0.74 (95% CI: 0.69, 0.78; Figure S1) for predicting the malignancy of ipsilateral BI-RADS 3 lesions.

**Table 2:** Clinicopathologic characteristics of 397 patients with newly diagnosed breast cancer who have additional ipsilateral ultrasound BI-RADS category 3 lesions

Characteristics	BI-RADS 3 lesions		*p*-value
	Benign (*n* = 358)	Malignant (*n* = 39)	
Age			0.07
< 40 y	89 (24.9)	15 (38.5)	
≥ 40 y	269 (75.1)	24 (61.5)	
Anatomic Location			< 0.001
Same quadrant	147 (41.1)	30 (76.9)	
Different quadrant	211 (58.9)	9 (23.1)	
Multiple primary cancers on US			0.80
No	315 (88.0)	34 (87.2)	
Yes	43 (12.0)	5 (12.8)	
BI-RADS of primary cancer on US			0.65
4A	36 (10.1)	6 (15.4)	
4B	118 (33.0)	13 (33.3)	
4C	108 (30.2)	9 (23.1)	
5	56 (15.6)	5 (12.8)	
6	40 (11.2)	6 (15.4)	
Maximum diameter of BI-RADS 3 lesions on US			0.50
≤ 1 cm	279 (77.9)	34 (87.2)	
> 1 cm and ≤ 2 cm	69 (19.3)	5(12.8)	
> 2 cm	10 (2.8)	0 (0.0)	
Maximum diameter of primary cancer on US			0.10
≤ 2 cm	180 (50.3)	22 (56.4)	
> 2 cm and ≤ 5 cm	163 (45.5)	13 (33.3)	
> 5 cm	15 (4.2)	4 (10.3)	
Pathologic characteristics of primary cancer			
Pathologic type			0.32
IDC	302 (84.4)	31 (79.5)	
DCIS	26 (7.3)	3 (7.7)	
ILC	14 (3.9)	4 (10.3)	
Others^#^	16 (4.5)	1 (2.6)	
Intrinsic subtypes^†^			0.15
HER2 positive	32 (9.6)	3 (8.1)	
Luminal A	53 (15.9)	6 (16.2)	
Luminal B HER2 +	60 (18.0)	2 (5.4)	
Luminal B HER2 −	162 (48.5)	25 (67.6)	
Triple negative	27 (8.1)	1 (2.7)	

Unless otherwise indicated, data are numbers of patients, and data in parentheses are percentages calculated by column

*US* ultrasound, *BI-RADS* breast imaging reporting and data system, *IDC* invasive ductal carcinoma, *DCIS* ductal carcinoma in situ, *ILC* invasive lobular carcinoma

^#^ Five cases of mixed invasive carcinoma, four cases of invasive carcinoma, three cases of mucinous carcinoma, three cases of invasive micropapillary carcinoma, and two cases of solid papillary carcinoma with invasion

^†^Data of intrinsic subtypes of the primary cancer were available in 371 patients

**Table 3:** Imaging characteristics of 397 primary cancers in patients with newly diagnosed breast cancer who have additional ipsilateral ultrasound BI-RADS category 3 lesions

Characteristics	BI-RADS 3 lesions		*p*-value
	Benign (*n* = 358)	Malignant (*n* = 39)	
Detailed ultrasonographic characteristics^#^			
Shape			0.67
Oval	18 (5.2)	3 (8.1)	
Round	5 (1.5)	0 (0.0)	
Irregular	321 (93.3)	34 (91.9)	
Orientation			0.07
Parallel	125 (36.3)	19 (51.4)	
Not Parallel	219 (63.7)	18 (48.6)	
Margin			0.26
Circumscribed	2 (0.6)	1 (2.7)	
Not circumscribed	342 (99.4)	36 (97.3)	
Echo pattern			0.24
Hypoechoic	322 (93.6)	33 (89.2)	
Isoechoic	2 (0.6)	0 (0.0)	
Mixed solid and cystic	8 (2.3)	3 (8.1)	
Heterogeneous	12 (3.5)	1 (2.7)	
Posterior features			0.16
No posterior features	316 (91.9)	32 (86.5)	
Enhancement	11 (3.2)	4 (10.8)	
Shadowing	15 (4.4)	1 (2.7)	
Combined pattern	2 (0.6)	0 (0.0)	
Calcification			0.94
No	193 (56.1)	21 (56.8)	
Yes	151 (43.9)	16 (43.2)	
Architectural distortion			0.03
No	339 (98.5)	34 (91.9)	
Yes	5 (1.5)	3 (8.1)	
Duct changes			0.69
No	328 (95.3)	35 (94.6)	
Yes	16 (4.7)	2 (5.4)	
Skin changes			0.10
No	344 (100.0)	36 (97.3)	
Yes	0 (0.0)	1 (2.7)	
Edema			NA
No	344 (100.0)	37 (100.0)	
Yes	0 (0.0)	0 (0.0)	
Vascularity			0.24
Absent	69 (20.1)	4 (10.8)	
Internal vascularity	220 (64.0)	24 (64.9)	
Vessels in rim	55 (16.0)	9 (24.3)	
Suspicious lymph node status			0.10
No	257 (71.8)	23 (59.0)	
Yes	101 (28.2)	16 (41.0)	
Mammographic characteristic^†^			
Suspicious calcification			0.002
No	232 (65.0)	15 (39.5)	
Yes	125 (35.0)	23 (60.5)	

Unless otherwise indicated, data are numbers of patients, and data in parentheses are percentages calculated by column

*BI-RADS* breast imaging reporting and data system, *NA* not available

^#^ Detailed ultrasonographic Characteristics of the primary cancer were available in 381 cases

^†^Mammographic characteristics were available in 395 cases

### Pathologic characteristics of patients with ipsilateral ultrasound BI-RADS 3 lesions

Among the 397 patients with ipsilateral ultrasound BI-RADS 3 lesions, pathologic results of the representative additional BI-RADS 3 lesions were available for 295 cases. Among 39 malignant BI-RADS 3 lesions, invasive ductal carcinoma (IDC) and ductal carcinoma in situ (DCIS) were equally common, each accounting for 41.0% (16/39). Fibroadenoma was the most frequent benign BI-RADS 3 lesion (49.6%, 127/256). In primary cancers, IDC was the most frequent malignancy (83.9%, 333/397), followed by DCIS (7.3%, 29/397). The detailed pathologic characteristics are presented in Tables 4 and 5.

**Table 4:** Patient-level pathology of primary breast cancers and representative additional ipsilateral ultrasound BI-RADS category 3 lesions

Lesions	Pathology	No. (%)
Primary cancer	Malignant	397 (100.0)
	IDC	333 (83.9)
	DCIS	29 (7.3)
	ILC	18 (4.5)
	Others^#^	17 (4.3)
Additional BI-RADS 3 lesions^‡^	Malignant	39 (13.2)
	IDC	16 (41.0)
	DCIS	16 (41.0)
	ILC	3 (7.7)
	Others^†^	4 (10.3)
	Benign	256 (86.8)
	Fibroadenoma	127 (49.6)
	Fibrocystic change	69 (27.0)
	ADH	18 (7.0)
	Adenosis	17 (6.6)
	Intraductal papilloma	6 (2.3)
	Radial scar	4 (1.6)
	Lobular neoplasia	2 (0.8)
	Columnar cell lesions	2 (0.8)
	Ductal adenoma	1 (0.4)
	Benign vascular lesions	1 (0.4)
	Mixed benign lesion*	9 (3.5)

Data are numbers of patients, and data in parentheses are percentages

*BI-RADS* breast imaging reporting and data system, *IDC* invasive ductal carcinoma, *DCIS* ductal carcinoma in situ, *ILC* invasive lobular carcinoma, *ADH* atypical ductal hyperplasia

^#^ Five cases of mixed invasive carcinoma, four cases of invasive carcinoma, three cases of mucinous carcinoma, three cases of invasive micropapillary carcinoma, and two cases of solid papillary carcinoma with invasion

^‡^ Pathologic results were unavailable for 102 ipsilateral ultrasound BI-RADS category 3 lesions, which were classified as benign based on ≥ 24-month imaging follow-up

^†^ Three cases of invasive carcinoma and one case of lymphovascular invasion

^*^Pathologic results include two or more benign histological types

**Table 5:** Patient-level correlation in pathology between primary cancers and additional malignant ipsilateral ultrasound BI-RADS category 3 lesions

Primary cancers	Additional malignant BI-RADS 3 lesions no. (%)			
	IDC	DCIS	ILC	Others^†^
IDC	16 (51.6)	12 (38.7)	0	3 (9.7)
DCIS	0	3 (100.0)	0	0
ILC	0	1 (25.0)	3 (75.0)	0
Others^#^	0	0	0	1 (100.0)

Data are numbers of patients, and data in parentheses are percentages calculated by row

*BI-RADS* breast imaging reporting and data system, *IDC* invasive ductal carcinoma, *DCIS* ductal carcinoma in situ, *ILC* invasive lobular carcinoma

^#^ One case of invasive carcinoma

^†^Three cases of invasive carcinoma and one case of lymphovascular invasion

## Discussion

In this large cohort of 1458 women with newly diagnosed breast cancer, we evaluated the malignancy risk of additional ultrasound BI-RADS category 3 lesions. At the patient level, 3.5% of women had malignant BI-RADS 3 lesions, with markedly higher risk for ipsilateral lesions, particularly those located in the same quadrant as the primary tumor. These findings indicate that the malignancy risk of additional BI-RADS 3 lesions in women with breast cancer exceeds the BI-RADS category 3 threshold of 2% and is strongly influenced by the anatomical proximity of these lesions to the primary tumor.

According to the ACR guideline, ultrasound BI-RADS 3 lesions are considered probably benign and are typically managed with short-term follow-up rather than immediate biopsy. However, determining the true malignancy risk of BI-RADS 3 lesions remains challenging, particularly in the preoperative setting [16, 17]. For women with newly diagnosed breast cancer, this uncertainty may complicate surgical planning, heighten psychological stress, and lead to delayed diagnosis if a lesion initially managed with surveillance subsequently proves malignant. This challenge is addressed in the 2025 BI-RADS update, which explicitly advises that category 3 should be avoided for findings on MRI in the setting of current breast cancer, suggesting that an upgrade to category 4 A may be warranted to facilitate prompt tissue diagnosis. Increasing evidence indicates that factors beyond imaging appearance should be taken into account when assigning a probably benign assessment [18]. Clinical characteristics such as older age, personal history of breast cancer, and the presence of additional breast cancer lesions have all been shown to be associated with a higher likelihood of malignancy of BI-RADS 3 findings [9, 10, 18–21]. Identifying high-risk additional ultrasound BI-RADS 3 lesions is crucial for tailored therapeutic strategies, particularly in selecting candidates for breast-conserving surgery [2, 22]. In addition, a detailed biological assessment of additional foci may influence the choice of adjuvant treatments [23, 24].

Previous studies have reported substantially elevated malignancy rates for additional ultrasound BI-RADS 3 lesions at the lesion level, with Kim et al and Lee et al reporting rates of 11.4% and 9.6%, respectively [9, 10]. Although the lesion-level malignancy rate in our cohort was lower (2.7%), it still exceeded the 2% BI-RADS category 3 benchmark. Consistent with the findings of Kim et al [10], our study also observed that the malignant risk increased with closer anatomical proximity to the primary cancer. Lesions in the same quadrant had the highest likelihood of malignancy, with a rate of 13.6% in our cohort compared with 21.2% reported by Kim et al [10]. Conversely, contralateral ultrasound BI-RADS 3 lesions showed a malignancy rate of only 0.9% (13/1,451) in our study, which is lower than the rates reported by Lee et al (10.1%) and Kim et al (4.2%) [9, 10], yet consistent with the current expert consensus [25]. These discrepancies may partly reflect the relatively small number of contralateral ultrasound BI-RADS 3 lesions evaluated in prior studies and their use of an earlier BI-RADS edition. In addition, our study utilized state-of-the-art ultrasound techniques with higher resolution, which facilitated the accurate identification of subtle yet critical features of malignancy that might otherwise be overlooked [11].

**Fig. 4:**
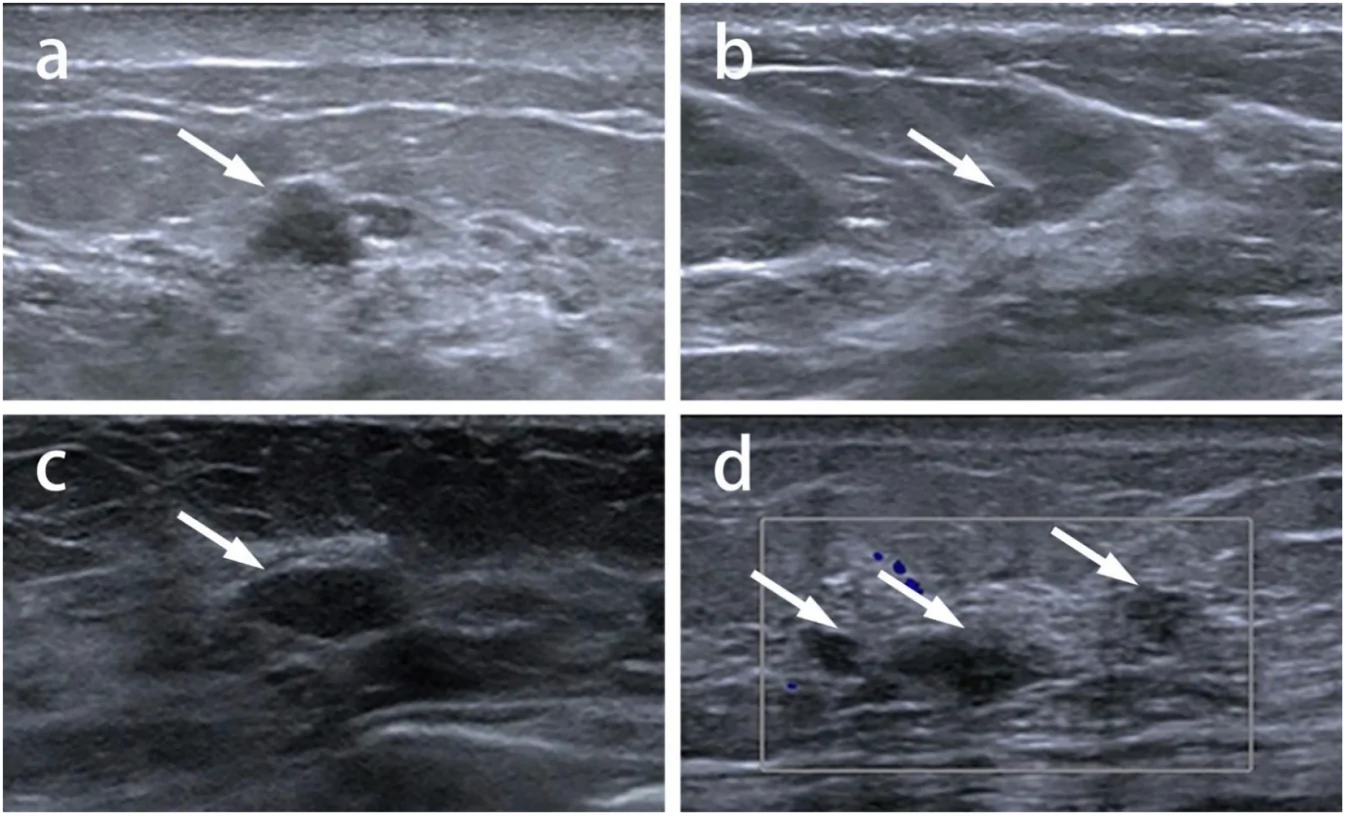
Representative false-negative cases of additional occult malignancies initially categorized as ultrasound BI-RADS category 3 lesions (selected from the 39 malignant cases detailed in Supplementary Table S1; underestimation reasons were classified by referencing the criteria described by Song SE et al [11]). **a** In a 61-year-old woman, breast US shows an irregular hypoechoic mass with a non-circumscribed margin (arrow), pathologically confirmed as IDC. The underestimation reason was attributed to “misinterpretation”. **b** In a 71-year-old woman, breast US shows an oval isoechoic mass measuring only 4 mm in diameter (arrow), pathologically confirmed as IDC. The underestimation reason was attributed to “small lesion size (< 5 mm)”. **c** In a 55-year-old woman, breast US shows a mass with an oval hypoechoic appearance and circumscribed margin (arrow), mimicking a benign mass, pathologically confirmed as IDC. The underestimation was attributed to its “benign appearance”. **d** In a 48-year-old woman, breast US shows multiple additional lesions (arrows), pathologically confirmed as DCIS. The underestimation reason was attributed to the presence of “multiple distracting lesions”. BI-RADS, breast imaging reporting and data system; US, ultrasound; IDC, invasive ductal carcinoma; DCIS, ductal carcinoma in situ

In our study, the identification of malignant ipsilateral ultrasound BI-RADS 3 lesions was particularly challenging. Notably, none of these malignancies exhibited sonographically visible microcalcifications. Furthermore, many of these lesions were small and exhibited only subtle suspicious features (Table S1 and Fig. 4). In such circumstances, it is particularly important to look for other predictors that cannot be ignored. Our study showed that the proximity of an additional BI-RADS 3 lesion to the primary lesion was an independent predictor for malignancy, consistent with previous findings [9, 10]. An anatomical relationship between additional lesion and primary cancer on preoperative breast MR imaging has also been identified as a discriminator of malignancy for additional ipsilateral lesions [26]. Notably, ultrasonographic architectural distortion of the primary cancer strongly predicted malignancy with an OR of 8.5 (95% CI: 1.6, 46.1; *p* = 0.01). Furthermore, suspicious calcification of the primary cancer on mammography was identified as another independent risk factor, consistent with findings from a small-cohort study demonstrating that the malignancy likelihood of additional breast ultrasound lesions was 25.0% (8/32) in patients without microcalcifications in the primary lesion and 36.8% (7/19) in those with microcalcifications [27]. While invasive lobular carcinoma (ILC) traditionally has a higher multiplicity risk than IDC [28], our study showed that ILC was not an independent risk factor, despite the highest malignant rate (22.2%, 4/18) of BI-RADS 3 lesions in primary breast cancer with ILC. This may be attributed to the limited number (*n* = 18) of patients with primary ILC and ipsilateral BI-RADS 3 lesions included in our study. Similarly, the primary tumor’s intrinsic subtype showed no significant correlation with the malignancy risk of additional BI-RADS 3 lesions.

In our study, the histopathology of malignant ipsilateral ultrasound BI-RADS 3 lesions showed a high degree of concordance with that of the corresponding primary cancers. Among the 32 malignant BI-RADS 3 lesions originating from the breast ductal system (IDC or DCIS), 31 (96.9%) shared the same ductal phenotype as the primary tumor. Likewise, all three BI-RADS 3 lesions diagnosed as ILC occurred in patients whose primary tumors were also ILC. Similar findings have been reported previously [29, 30]. Kanumuri et al [30] observed a high rate of histologic concordance between the concurrent lesions, with 97.4% (189/194) for ductal-associated cancers and 78.8% (26/33) for lobular-associated cancers.

Our study had several limitations. First, the retrospective single-center design and availability of data introduced selection bias. Pathologic and follow-up data for certain ultrasound BI-RADS 3 lesions were unavailable due to mastectomy. Second, because preoperative breast MRI was performed in fewer than half of the patients and no definite suspicious correlate was identified for additional BI-RADS 3 lesions, MRI findings were not systematically incorporated into the analysis. Third, important variables such as family history, germline mutation status (e.g., BRCA1/2), and ultrasonographic elastography findings were not available. Fourth, although the 2025 BI-RADS update was released during the revision, we maintained the 2013 standard to ensure methodological consistency with our original study design. Future investigations should focus on prospective, multicenter studies that incorporate the latest BI-RADS descriptors, comprehensive imaging, clinical, pathologic, and genetic data to better delineate the clinical relevance, biological heterogeneity, and optimal management strategies for additional ultrasound BI-RADS 3 lesions in patients with newly diagnosed breast cancer.

In conclusion, our study demonstrated that additional ipsilateral ultrasound BI-RADS category 3 lesions exhibited a substantially high likelihood of malignancy, especially when located in the same quadrant as the primary tumor, and biopsy should be recommended. Conversely, additional contralateral lesions showed a low malignancy rate; when clinical and family histories are considered together, a conservative follow-up strategy remains appropriate. The malignancy of additional ipsilateral ultrasound BI-RADS 3 lesions was associated with the same-quadrant location as the primary lesion, ultrasonographic architectural distortion, and mammographic suspicious calcification of the primary lesion.

## Supplementary information

ELECTRONIC SUPPLEMENTARY MATERIAL

## Data Availability

The datasets generated and analyzed during the current study are not publicly available due to data protection and security policies but are available from the corresponding author on reasonable request.

## Abbreviations

ACR: American College of Radiology
BI-RADS: Breast imaging reporting and data system
DCIS: Ductal carcinoma in situ
IDC: Invasive ductal carcinoma
ILC: Invasive lobular carcinoma
IQR: Interquartile range
OR: Odds ratio
VAB: Vacuum-assisted biopsy
