# Supplementary material for: The underestimated risk of additional ipsilateral BI-RADS 3 lesions identified by preoperative ultrasound in breast cancer patients
Source: Insights Imaging. 2026 Oct 16;17:263. doi: 10.1186/s13244-026-02366-6 (PMC13675164; doi:10.1186/s13244-026-02366-6)
Supplement: Supplementary file 1 [file 13244_2026_2366_MOESM1_ESM.pdf]

The Underestimated Risk of Additional Ipsilateral BI-RADS 3 Lesions Identified by Preoperative Ultrasound in Breast Cancer Patients

ELECTRONIC SUPPLEMENTARY MATERIAL

Insights Imaging (2026) Hu Y, Wang H, Wu M et al.; DOI:

Table S1: Characteristics of 39 cases with malignant ipsilateral ultrasound BI-RADS category 3 lesions

Figure S1: Graph shows diagnostic performance of different characteristic(s) for predicting malignancy of additional ultrasound BI-RADS category 3 lesions. The *P* value associated with the area under the curve (AUC) refers to the combination of three characteristics' ability to discriminate between benign and malignant category 3 lesions. BI-RADS = Breast Imaging Reporting and Data System, US = ultrasound, MG = mammography.

Table S1: Characteristics of 39 cases with malignant ipsilateral ultrasound BI-RADS category 3 lesions

Case (no.)	Age (years)	Anatomic relationship between category 3 and primary lesion	Architectural distortion of primary lesion on US#	Suspicious calcification of primary lesion on MG#	Max. diameter of primary lesion (cm)	Max. diameter of category 3 lesion (cm)	Pathology of primary lesion	Intrinsic subtype of primary lesion#	Pathology of category 3 lesion	Intrinsic subtype of category 3lesion#	Underestimation reason of category 3 lesion*	Surgery therapy
1	38	Different quadrant	No	No	5.4	0.7	IDC	Luminal B HER2+	IDC	Luminal B HER2+	d	Mastectomy + ALND
2	48	Same quadrant	No	Yes	2.1	1.0	IDC	Luminal B HER2-	DCIS	NA	d	Mastectomy + ALND
3	65	Same quadrant	No	Yes	1.7	0.7	IDC	Luminal B HER2-	IDC	Luminal B HER2-	b	Mastectomy + ALND
4	61	Same quadrant	No	Yes	2.5	1.3	IDC	Luminal B HER2-	DCIS	NA	a	BCS + SLNB
5	51	Same quadrant	No	Yes	1.4	0.9	DCIS	NA	DCIS	NA	a	Mastectomy + ALND
6	55	Same quadrant	Yes	Yes	2.1	1.1	IDC	Luminal B HER2-	IDC	NA	c	Mastectomy + ALND
7	36	Same quadrant	Yes	Yes	1.1	0.6	ILC	Luminal B HER2+	ILC	NA	c	Mastectomy + ALND
8	42	Same quadrant	NA	NA	2.3	0.8	ILC	Luminal B HER2-	ILC	NA	c	Mastectomy + SLNB
9	41	Same quadrant	No	No	1.4	0.6	IDC	Luminal B HER2-	DCIS	NA	d	BCS + SLNB
10	43	Same quadrant	No	No	2.0	0.7	ILC	Luminal A	DCIS	NA	c	Mastectomy + SLNB
11	59	Different quadrant	No	Yes	1.8	0.9	IDC	NA	DCIS	NA	d	Mastectomy + SLNB
12	42	Different quadrant	No	Yes	1.8	0.5	IDC	Luminal B HER2-	IDC	Luminal B HER2-	b	BCS + ALND
13	29	Same quadrant	No	Yes	1.7	0.4	IDC	Luminal B HER2-	IDC	NA	b	BCS + SLNB

14	39	Same quadrant	No	Yes	2.0	0.8	IDC	Luminal B HER2-	DCIS	NA	d	BCS + SLNB
15	31	Same quadrant	No	Yes	4.8	1.0	IDC	Luminal B HER2-	IDC	Luminal B HER2-	c	Mastectomy + ALND
16	46	Different quadrant	No	Yes	1.9	0.7	IDC	Luminal A	DCIS	NA	d	Mastectomy + ALND
17	41	Same quadrant	No	No	1.7	1.0	DCIS	Luminal B HER2-	DCIS	NA	a	BCS + SLNB
18	38	Same quadrant	NA	No	0.8	0.5	IDC	Luminal B HER2-	DCIS	NA	b	BCS + SLNB
19	51	Same quadrant	No	No	5.3	0.8	Invasive carcinoma	HER2+	Lympho-vascular invasion	NA	c	BCS + SLNB
20	42	Same quadrant	No	Yes	2.4	0.5	IDC	Luminal A	DCIS	NA	b	BCS + SLNB
21	33	Same quadrant	No	Yes	5.0	0.9	IDC	Luminal B HER2-	IDC	Luminal B HER2-	c	Mastectomy + ALND
22	46	Same quadrant	No	Yes	1.0	0.5	IDC	Luminal B HER2-	IDC	Luminal B HER2-	b	BCS + SLNB
23	71	Same quadrant	No	Yes	1.3	0.4	IDC	Luminal A	IDC	NA	b	BCS + SLNB
24	39	Same quadrant	No	No	2.0	0.7	ILC	Luminal B HER2-	ILC	NA	d	Mastectomy + ALND
25	47	Different quadrant	No	No	1.5	0.5	IDC	Luminal B HER2-	IDC	NA	c	Mastectomy + SLNB
26	46	Same quadrant	No	No	1.7	1.1	IDC	HER2+	IDC	HER2+	a	Mastectomy + SLNB
27	44	Different quadrant	No	No	2.6	0.6	IDC	Luminal A	DCIS	NA	d	Mastectomy + SLNB
28	35	Same quadrant	No	Yes	1.2	0.7	IDC	Luminal B HER2-	DCIS	NA	c	Mastectomy + SLNB
29	31	Same quadrant	No	No	3.4	1.0	DCIS	Luminal B HER2-	DCIS	NA	a	Mastectomy + SLNB
30	30	Same quadrant	No	Yes	5.6	0.8	IDC	Luminal B HER2-	Invasive carcinoma	NA	c	Mastectomy + ALND
31	32	Same	No	Yes	2.7	0.5	IDC	HER2+	Invasive	Luminal B	c	Mastectomy

		quadrant							carcinoma	HER2+		+ ALND
32	33	Same quadrant	No	No	3.8	1.3	IDC	Triple negative	IDC	NA	c	BCS + SLNB
33	48	Same quadrant	No	No	1.9	0.9	IDC	Luminal B HER2-	IDC	Luminal B HER2-	d	BCS + ALND
34	61	Different quadrant	Yes	Yes	1.9	1.1	IDC	Luminal B HER2-	IDC	Luminal B HER2-	a	Mastectomy + ALND
35	45	Different quadrant	No	No	2.5	0.7	IDC	Luminal A	DCIS	NA	d	Mastectomy + SLNB
36	29	Same quadrant	No	Yes	5.8	0.7	IDC	Luminal B HER2-	Invasive carcinoma	NA	c	Mastectomy + ALND
37	41	Different quadrant	No	Yes	1.7	0.4	IDC	Luminal B HER2-	IDC	Luminal B HER2-	b	BCS + ALND
38	33	Same quadrant	No	Yes	4.6	1.0	IDC	Luminal B HER2-	IDC	Luminal B HER2-	c	Mastectomy + ALND
39	43	Same quadrant	No	No	1.3	0.6	IDC	Luminal B HER2-	DCIS	NA	d	BCS + SLNB

Note. — BI-RADS = Breast Imaging Reporting and Data System, US = ultrasound, MG = mammography, IDC = invasive ductal carcinoma, DCIS = ductal carcinoma in situ, ILC = invasive lobular carcinoma, NA = not available, ALND = axillary lymph node dissection, BCS = breast-conserving surgery, SLNB = sentinel lymph node biopsy.

# Some data is not available.

* The reasons for the underestimation of BI-RADS category 3 lesions were based on the criteria described by Song SE et al. [(1)]: (a) misinterpretation, (b) small lesion size (<5 mm), (c) benign appearance, (d) multiple distracting lesions (when at least three circumscribed benign-looking solid masses were present in the ipsilateral breast), (e) slow-growing lesions (when lesion stability was confirmed during 24 months of follow-up), (f) missed core biopsy (when a malignant lesion had been sampled with core needle biopsy and the result was benign), or (g) lesions mimicking postoperative scars. When two or more reasons existed, the most influential reason was selected for the case.

1. Song SE, Cho N, Chu A, et al. Undiagnosed Breast Cancer: Features at Supplemental Screening US. Radiology 2015;277(2):372-380. doi: 10.1148/radiol.2015142960

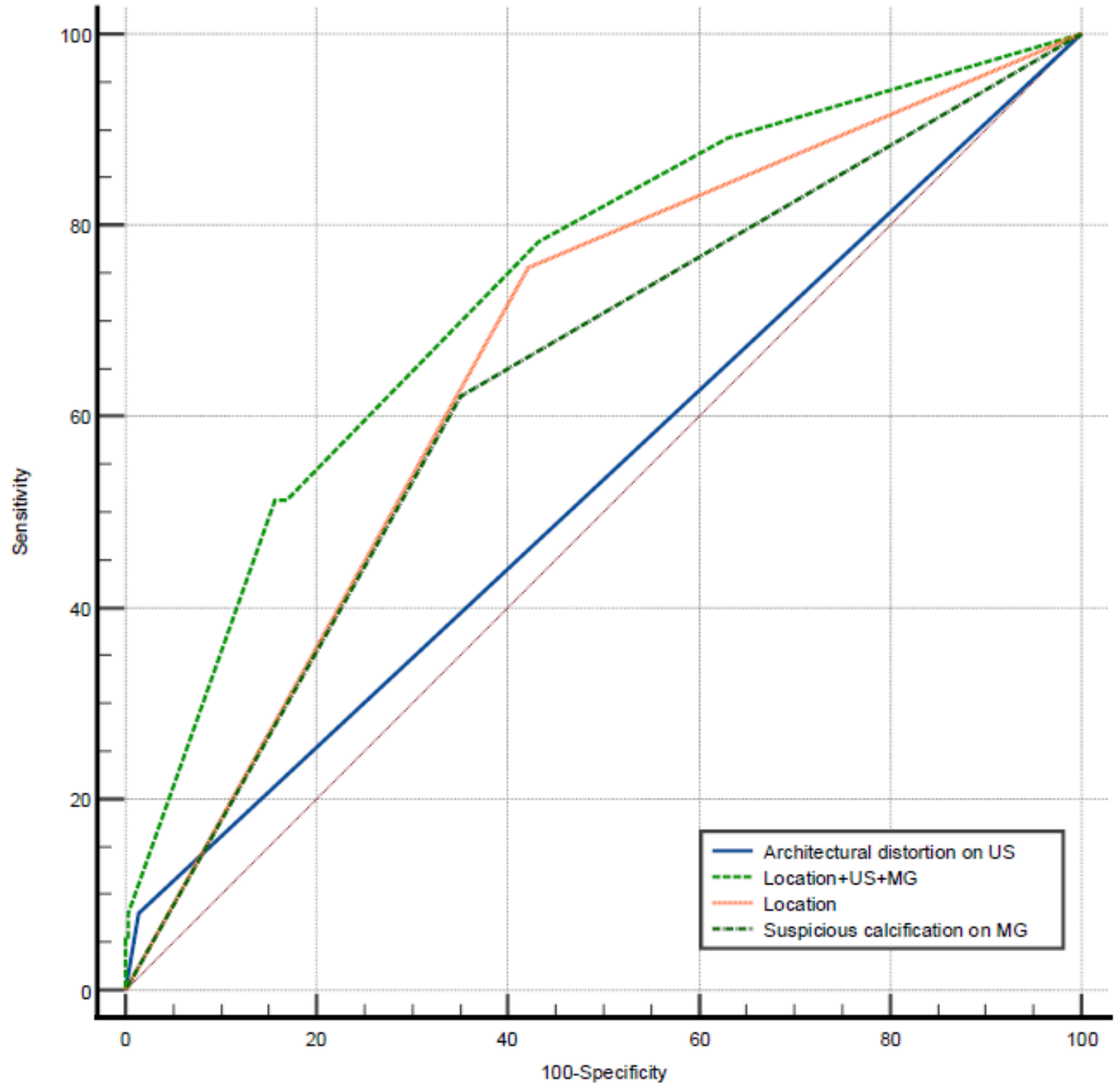

	AUC	95% CI	*P*-value
Location + US + MG	0.74	0.69 to 0.78	/
Location	0.67	0.62 to 0.72	0.01
Architectural distortion on US	0.53	0.48 to 0.58	< 0.001
Suspicious calcification on MG	0.64	0.58 to 0.68	0.009

Figure S1: Graph shows diagnostic performance of different characteristic(s) for predicting malignancy of additional ultrasound BI-RADS category 3 lesions. The *P* value associated with the area under the curve (AUC) refers to the combination of three characteristics' ability to discriminate between benign and malignant category 3 lesions. BI-RADS= Breast Imaging Reporting and Data System, US = ultrasound, MG = mammography.
